# Multi-dimensional computational pipeline for large-scale deep screening of compound effect assessment: an in silico case study on ageing-related compounds

**DOI:** 10.1038/s41540-019-0119-y

**Published:** 2019-11-26

**Authors:** Vipul Gupta, Alina Crudu, Yukiko Matsuoka, Samik Ghosh, Roger Rozot, Xavier Marat, Sibylle Jäger, Hiroaki Kitano, Lionel Breton

**Affiliations:** 1 The Systems Biology Institute, Tokyo, Japan; 2 L’Oréal Research and Innovation, Aulnay-sous-Bois, France; 3 Okinawa Institute of Science and Technology, Okinawa, Japan

**Keywords:** Target identification, Virtual drug screening, Software, Biochemical networks

## Abstract

Designing alternative approaches to efficiently screen chemicals on the efficacy landscape is a challenging yet indispensable task in the current compound profiling methods. Particularly, increasing regulatory restrictions underscore the need to develop advanced computational pipelines for efficacy assessment of chemical compounds as alternative means to reduce and/or replace in vivo experiments. Here, we present an innovative computational pipeline for large-scale assessment of chemical compounds by analysing and clustering chemical compounds on the basis of multiple dimensions—structural similarity, binding profiles and their network effects across pathways and molecular interaction maps—to generate testable hypotheses on the pharmacological landscapes as well as identify potential mechanisms of efficacy on phenomenological processes. Further, we elucidate the application of the pipeline on a screen of anti-ageing-related compounds to cluster the candidates based on their structure, docking profile and network effects on fundamental metabolic/molecular pathways associated with the cell vitality, highlighting emergent insights on compounds activities based on the multi-dimensional deep screen pipeline.

## Network pharmacology: merging the boundaries of network- and structural biology

The ability to study the precise effect of chemical compounds on specific molecular entities plays a crucial role in understanding their safety and efficacy landscapes. Towards this goal, an international team of researchers led by Lionel Breton and Hiroaki Kitano proposed an innovative multi-dimensional computation pipeline for large-scale assessment of chemical compounds. The pipeline harnesses the benefits of both structural and network biology together to cluster chemicals on their network effects across pathways and molecular interaction maps. Further, the pipeline was applied to screen a set of anti-ageing-related compounds on fundamental metabolic/molecular pathways associated with the cell vitality. The results highlight the importance of network-based multi-dimensional screens in capturing emergent properties of compounds which may not be apparent from a single-dimensional analysis such as molecular docking or chemical similarity.

## Introduction

Developing cutting-edge methodologies for assessing and optimising the efficacy of chemical compounds is a challenge for developing a 21st-century paradigm in compound screening. While the conceptual framework of 20th-century assessment studies was dominated by animal experiments, recent developments in experimental and computational techniques provide alternative opportunities to gain a systems-level understanding of the underlying biology driving the effects of chemicals on humans.^1–3^ Particularly, the ability to study the precise effect of chemical compounds on specific molecular entities plays a crucial role in understanding their toxicological and efficacy landscapes.^4,5^

Systems-oriented, network pharmacology-based approaches combining multiple dimensions of the compound structure, functions and molecular networks, unravel a unique opportunity for developing computational pipelines that provide the capability to “deep screen” compounds on different axes of biology to obtain mechanistic insights into their effects.

Recent advancements in in silico techniques, such as structural biology, molecular docking, molecular pathway building and computational chemistry, supply the community with sophisticated tools for predicting the effect of protein–drug interaction at phenotypic level.^6–8^ Further, advancements in the field of protein–protein interaction, pathway analysis and literature-mining allow large-scale-free networks to assist the decision making based on the valuable information about biological perturbation generated from these sources.^5,8–13^ Each of the above approaches provides a specific perspective to look for relationships within biological processes. However, the complexity of the molecular interactions at the cellular level and the potential for collateral interactions entail the development of methodologies that can connect the different perspectives and obtain deeper, emergent insights into the compound effect landscape.^14^

For example, while state-of-the-art docking tools provide simulation results for potential binding scores of compounds to known targets or proteins, each tool has its unique advantages and drawbacks. To obtain a high-precision score, it is crucial to building a computational framework that can leverage the advantages of specific tools, while reconciling their inherent limitations, as demonstrated by the *systemsDock* system for network pharmacology-based prediction and analysis of molecules.^15,16^ Further, to comprehend the impact of the docking of compounds to targets, it is important to study their effects holistically, at the level of molecular interactions rather than individual targets. Such system level studies can provide more profound insights into the potential mode of action (MOA) of specific compounds and identify network effects on safety and efficacy.

In this framework, the article proposes an innovative computational pipeline for large-scale assessment of chemical compounds to generate testable hypotheses on the pharmacological landscapes as well as new mechanisms of efficacy on phenomenological processes. Specifically, it analyses and clusters chemical compounds based on multiple dimensions—structural similarity, binding profiles and, more importantly, their network effects across pathways and molecular interaction maps. Further, to demonstrate the ability of the pipeline to obtain deeper, mechanistic insights into compound effects, we apply the pipeline on a screen of compounds to cluster the candidates based on their structure, docking profile and network effects on fundamental signalling/metabolic pathways associated with metabolic and cellular stress, damage and/or other factors that directly or indirectly affect the cell vitality.

In the next section, we provide a detailed outline of the pipeline, followed by discussions on the application of the pipeline featuring a case study for screening chemicals on important molecular/metabolic pathways for cell vitality and conclude with discussions on the challenges and opportunities of building deep screening pipelines for compound assessment.

## Results

### Overview: network-based compound screening pipeline

**Fig. 1:**
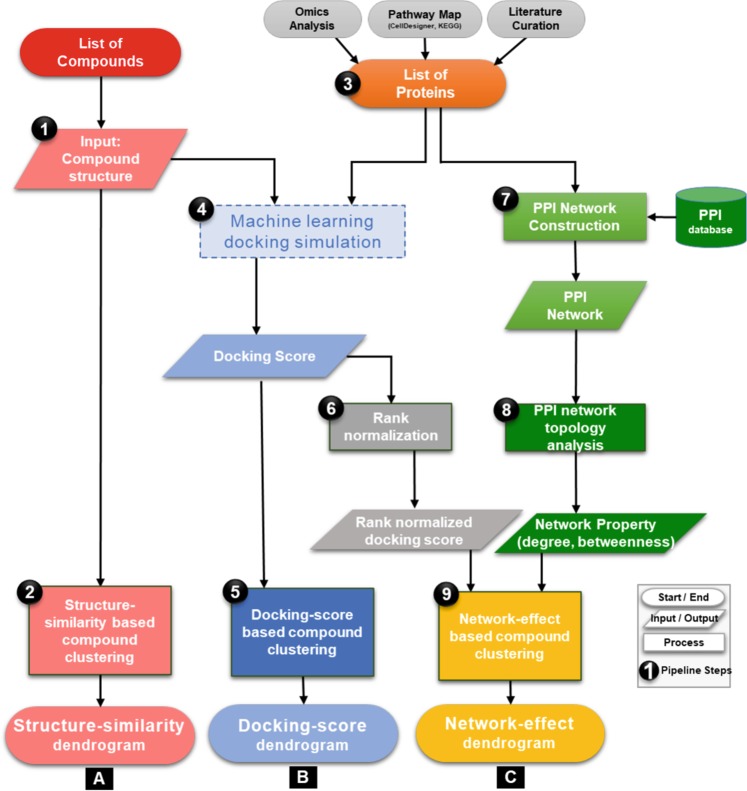
Flowchart representing the basic workflow of network-based compound screening pipeline. Each of the steps described in the text is marked in black circles. Input/output, process and start/end are described by proper flowchart symbols.

This section outlines the new network-based screening pipeline as illustrated in Fig. 1 and Box 1, along with the detailed methodology in each step of the pipeline. We systematically elucidate each step of the pipeline, highlighting the inputs and outputs and associated analysis of the computational flow.

#### 1. Input test compounds

A list of test compounds and associated structure files in “*sdf*” or mol or SMILES format are used as input to the pipeline.

#### 2. Structure-similarity-based compound clustering

To determine the landscape of the compound structures, a structure-similarity analysis is performed using a flexible maximum common substructure (FMCS) algorithm. The FMCS algorithm is an improved version of maximum common substructure (MCS) search method that allows small mismatches during the structure comparison.^17^ This has an advantage over MCS, as it results in the identification of more common substructure and provided higher sensitivity.^17^ The FMCS algorithm is also efficient at identifying local structural similarities between chemicals with significant differences in molecule size. Similarities between compounds are measured as Overlap coefficient defined as ***n/min(c1,c2)*** where ***n*** is the number of atoms in the MCS, ***c1*** and ***c2*** are number of atoms in the input compounds.^18^ The pipeline computes an overlap coefficient matrix for all input compounds and performs clustering of compounds using “pvclust” function and correlation-based dissimilarity matrix as available in R package.^19^ R function “pvclust” performs hierarchical cluster analysis by calculating AU (Approximately Unbiased) *p*-value using multiscale bootstrap resampling.^19^ Finally, the pipeline generates a structure-similarity dendrogram plot for visualisation.

#### 3. Protein input data preparation

Another input for the pipeline is a list of proteins related to a target biological phenomenon. Our pipeline is designed to provide the user with the flexibility to input either a list of proteins or whole pathway maps, associated with the biological phenomenon. The protein list can be obtained by using the following, non-exhaustive list of options: (1) genes/proteins identified in omics data analysis, (2) biomedical literature curation or (3) pathway curation. In this study, as we focus on finding chemicals with similar effects on cell vitality, we built a molecular mechanistic pathway map of its associated relevant molecular/metabolic pathways by literature curation, then identified the list of proteins for compounds screening as described in the case-study section of this manuscript.

#### 4. Machine-learning-based docking simulation

After preparing the input proteins and compound list, the pipeline performs docking simulation to generate docking scores for each set of protein–compound pairs in the input using *systemsDock* web-service.^16^ *systemsDock* rapidly and efficiently calculates the binding potential of a small molecule, such as a drug or candidate molecule, to a set of target proteins. It takes advantage of the multiple docking tools and uses their outputs to train a machine-learning model to obtain accurate prediction scores for the docking simulations. Its ability to integrate and learn from multiple state-of-the-art algorithms allows the system to predict at high-precision accuracy compared to the individual docking simulation systems both commercial (GOLD,^20^ eHiTs^21^) and academic-free versions (Vina^22^). Benchmark validation studies on *systemsDock* have demonstrated its ability to predict, with high accuracy, the primary targets of kinase inhibitors when compared to other off-the-shelf techniques.^15^

Both compound(s) structure file and protein-list/pathway maps are uploaded to the *systemsDock* server. For each of the target proteins in the list or pathway map, *systemsDock* automatically searches for the available tertiary structures in RCSB protein data bank^23^ and then retrieves the best resolution structure using a local synced copy of RCSB PDB database.^16^ Next, the binding site, if any, is assigned to the most prominent native ligand in the co-crystallised complex structure. Notably, this is a critical step in the pipeline where each assigned binding site is manually checked, as sometimes the protein structures might contain glycerol or detergent or some other kind of crystallography artifacts. Because of ligand like properties, these might also be assigned as the binding site that eventually will lead to false positive results. Thus, to maintain the quality of the analysis, binding site was assigned after manually checking each protein structure. Finally, docking is performed on *systemsDock* server that generates docking score in the range 0–10, representing the negative logarithm of the experimental dissociation/association constant (*pK*_*d*_*/pK*_*i*_), by evaluating single best most reliable binding pose between each protein–compound pair as described previously.^15,16^

#### 5. Docking Score-based compound clustering

Next, the docking scores computed for each of the compound–protein pairs are downloaded from the server and are transformed in the form of a docking-score matrix. To further comprehend the similarities/differences in the docking profile between compounds, the pipeline performs docking score-based hierarchical clustering of compounds using the docking score matrix and generates a dendrogram plot for visualisation, as described in step 2.

#### 6. Docking score rank normalisation

Docking score is generally biased by the additive nature of enthalpic effects associated with increasing compound size.^24^ As the current pipeline aims to screen a diverse set of chemicals for their overall network-level effects, it is important to normalise the docking scores to efficiently capture the unbiased effect of compound-protein interaction over the network. Docking scores generated in the previous step are normalised using *rank()* function in R by replacing docking scores with a rank in descending order starting with 1 for the highest docking score for a compound against all proteins. Rank ties were solved by computing and assigning the average rank (Box 1). While the technique has been applied previously to microarray datasets to reduce the technological noises,^25–27^ here it is used to generate a docking profile of compound against all proteins for subsequent analysis.

#### 7. Protein–protein interaction (PPI) network construction

While molecular interaction pathways provide a potential molecular mechanistic interaction for the constituents, their coverage can be limited by the knowledge of biochemical interactions. Large-scale PPI maps provide a basic abstraction of larger complex pathways that control the major cellular and molecular machinery determining the disease or healthy state of an organism. Hub protein nodes with higher degree of interactions in the PPI network represent the key targets drugging which, leads to a substantial effect on the cellular machinery. While docking provides an insight into the chemical-protein interactions, PPI network was used to compute the importance of each protein target used for docking in the previous step. In this pipeline, PPI network was generated from the STRING (http://string-db.org/) database^28^ that provides known PPI curated from published sources such as high-throughput experiments, co-expression, genomics and literature search. The initial list of target proteins used for docking is used as input in the STRING database to generate a PPI network for human isoforms with a high confidence score of 0.7.^28^ In STRING, confidence scores are used to establish the probability of interaction between two proteins based on the authenticity of the source of interaction.

#### 8. Network topology analysis

Using the NetworkAnalyzer function in Cytoscape,^29^ the network specific parameters, such as node degrees, betweenness, etc. that specify the dynamics of a network, are calculated. In this pipeline, we used node degree parameter that represents the number of connections that each node (protein) makes with other nodes (proteins) in the PPI network.

#### 9. Network-effect based compound clustering

To capture the network level effect of compound docking on PPIs, the analysis is focused on key network parameter like degree (i.e., number of interactions for a specific protein or molecular entity). Rank normalised docking score for each target protein is multiplied by its node degree parameter, amplifying the effect of compound binding. Finally, compounds are clustered using hierarchical clustering and a dendrogram is generated for visual inspection, as described in step 2.

The pipeline provides multiple clustering outputs [structural-, docking- and network-based clustering] with progressively deeper dimensions of the biology being integrated into the various steps of the pipeline, thereby providing a comparative view of the compound effect as captured by the pipeline. The intermediate outputs, structural- and docking-based clusters, provide an overview of chemical similarities and interaction landscapes but fail to capture the effect of these interactions at the phenomenon level. In contrast, the final output, network-effect based clustering elucidates the effects of each chemical beyond the dimensions of structure or target binding affinity at the phenotypic level.

In the next section, we provide a more detailed analysis of the comparative clustering results for the specific use case on anti-ageing-related compounds. It is pertinent to mention here that, while the current pipeline is customised to provide clustering outputs, the overall computational framework can also provide rank-order of compounds for use cases involving prioritisation of candidates for application in drug discovery phases.

### Case study: application of the pipeline on an ageing-related compound screening

To assess the performance of the pipeline, we present the case study on an ageing-related compound screen that analyses and clusters the candidate compounds based on their multi-dimensional effects on indispensable metabolic and molecular pathways associated with cell vitality. Deterioration of these pathways results in the dysregulation of the molecular machinery that contributes to the progressive time-dependent loss in cellular and tissue integrity, which characterises fundamental biological phenomena such as ageing.^30–39^ Designing interventions that can revert the effect of perturbations in these pathways will significantly benefit researchers in identifying new agents for healthy ageing and reduce healthcare expenses. Several studies that have used different strategies to identify compounds with potential anti-ageing properties.^40–42^ However, to our current knowledge, there are no studies that have built a comprehensive molecular mechanistic pathway map of cell vitality-associated pathways and used it as a base for clustering compounds.

We outline the inputs and the processes associated with the pipeline, specifically highlighting the role of the multi-dimensional pipeline in identifying deeper insights into the effect of the compounds (rapamycin and vitamin C in one case and retinol and retinoic acid in another).

### The input to the pipeline

#### Test compounds

**Fig. 2:**
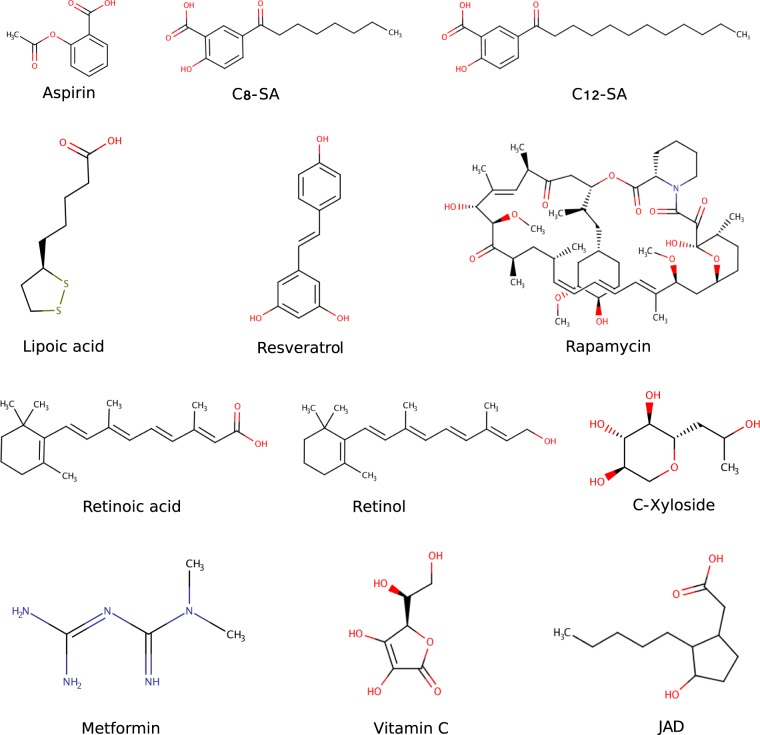
Twelve test compounds with different sizes and properties are used in the case study of network-based compound screening pipeline. Some of the compounds are known to have significant anti-ageing properties.

Twelve chemical compounds with different molecular weights, physico-chemical properties and known or unknown mechanisms of action were selected (Fig. 2, Supplementary Table S1) for a proof of concept study. Several compounds in the list, such as vitamin C, retinol, retinoic acid, resveratrol, LR2412/JAD, c-xyloside (Proxylane^TM^) were reported to affect skin ageing parameters.^43–51^ Rapamycin, metformin, resveratrol, C8-SA, acetyl salicylic acid, salicylic acid have also been described as longevity compounds in model organisms.^42,50,52–61^

#### Constructing pathway map

**Fig. 3:**
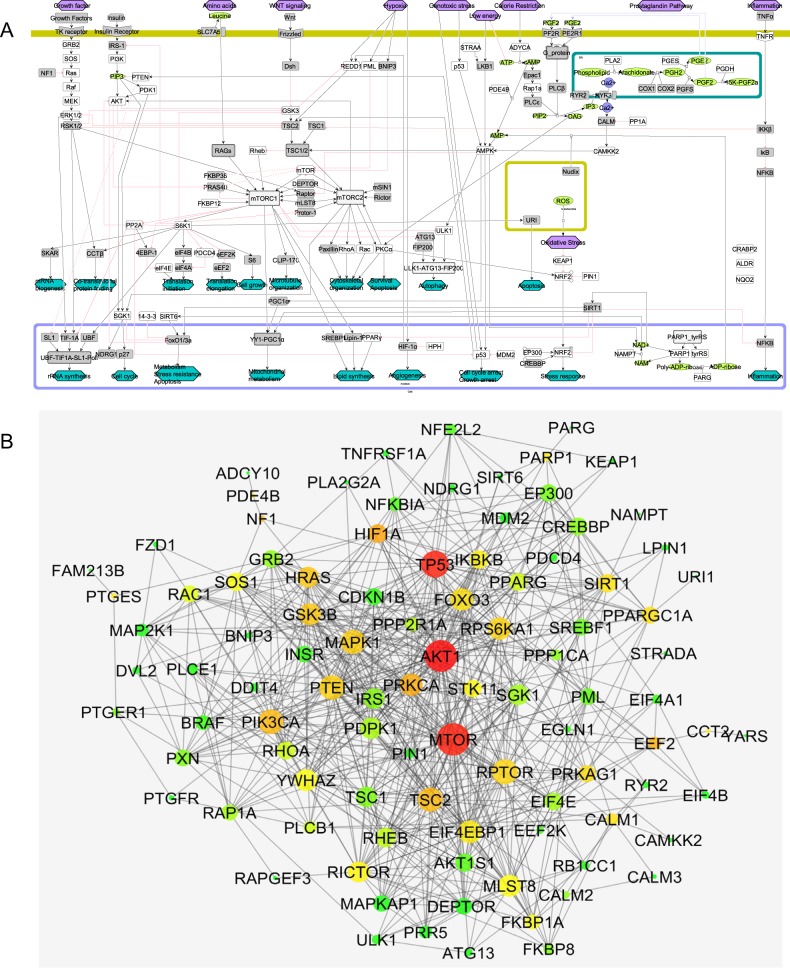
Construction of pathway map and PPI interaction map. **a** Molecular mechanistic pathway map of signalling and metabolic pathways associated with cell vitality were manually curated and constructed on CellDesigner 4.3. The map includes all the important species (protein, complexes, metabolite, DNA, RNA) and cellular compartments (such as mitochondria, nucleus and ER). **b** Protein–protein interaction (PPI) network was generated using STRING database for the proteins in the pathway map. The current visualisation was generated using Cytoscape, with larger node representing high degree and vice versa. Similarly, low to high betweenness centrality of the node in the PPI network was highlighted between green–yellow–red.

To build a molecular mechanistic map of cell vitality-associated pathways, we collected literature (published articles, reviews; compiled in Supplementary Table S2) available in public domain. This information was used to manually curate and build the map with CellDesigner 4.3.^62,63^ A score of proteins was mapped from relevant published data to capture the important pathways associated with maintaining cell viability (Fig. 3a). Precisely, a potential set of proteins include key components around fundamental signalling and metabolic pathways (energy balance, metabolism, mitochondria, oxidative stress-related molecules—FOXO3, AMPK, NRF2, mTOR, PGC-1alpha, PDE4, etc.).^64–71^ The map includes a total of 179 species connected by 214 reactions. Under species, there are 116 proteins, 11 complex and 20 simple molecules. Nine different signals (stimuli) are represented in the maps including genomic stress, low energy, hypoxia, growth factor, inflammation, caloric restriction and prostaglandin pathway. All proteins were mapped in a top-to-bottom approach where the top part represents the signalling and the bottom represents the downstream effects. Also, based on the literature search, 19 different downstream effects that are related to cellular integrity and vitality have been mapped (Fig. 3a).^30,32,72–77^

#### PPI network

Next, a PPI network for the proteins that are part of the above pathway map was built using STRING database (Fig. 3b; Supplementary Table S3 sheet “Pathway Map Protein”). The PPI network contains 106 protein nodes and 759 connections (Supplementary Table S3 sheet “PPI network”). A network topology parameter, node degree was generated for the PPI network (Supplementary Table S3 sheet “PPI Degree”) and included in network-based compound clustering step.

### Outputs from the pipeline

#### Structure-similarity-based compound clustering

**Fig. 4:**
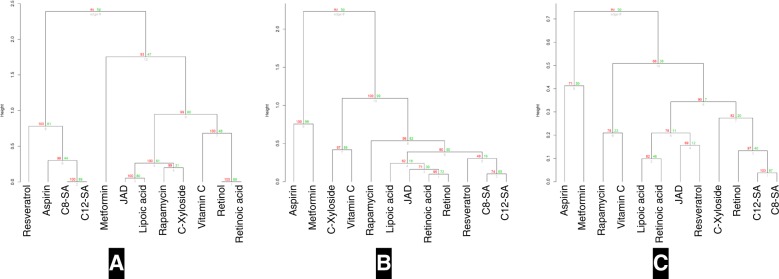
Compound clustering dendrograms for (**a**) structural-similarity, (**b**) docking-score and (**c**) network-effect.

To understand the difference in structural landscape and identify chemically more meaningful information on compound similarity, a structure-similarity analysis was performed using FMCS algorithm integrated into R,^17^ and visualised using dendrogram (Fig. 4a). All compounds except metformin formed a large group of structurally similar isolates. As expected, structurally similar compounds such as retinoic acid and retinol, C8-SA and C12-SA are clustered together. Interestingly, C-xyloside and rapamycin that differ significantly in size were also paired together. After careful analysis, we found that rapamycin contains a small signature of C-xyloside in its huge structure (Supplementary Figure S1). We further tested the Morgan or Extended Connectivity fingerprints (ECFPs) that represent a molecular structure using topological atom neighbourhoods. The clustering results obtained using ECFPs are similar to the FMCS based clustering results (Fig. 4a and Supplementary Figure S2). Surprisingly, other conventional methods such as MACCS-based fingerprints failed to identify these significant similarities between these two compounds (data not shown).

#### Docking-score based compound clustering

Out of 116 proteins present in the pathway map, structure for 45 proteins (~40%) with a resolution less than 3 angstroms and a well-defined binding pocket could be identified (Table 1). Docking was performed using *systemsDock* and docking scores were generated for selected 45 proteins against all test compounds (Supplementary Table S4 sheet “Docking Score”). Test compounds were clustered over docking score and visualised by dendrogram (Fig. 4b).

**Table 1:** Table showing the list of proteins in the pathway map, associated tertiary structures used for docking simulation, and the node degree computed from the PPI network.

Symbol	PDB ID	PDB resolution (Å)	PPI network node degree
14-3-3	4IHL	2.2	27
ADYCA	4CLT	1.95	1
AKT	4GV1	1.49	53
AMPK	2UV4	1.33	20
CAMKK2	2ZV2	2.4	3
eIF4E	4TPW	1.5	18
EP300	3BIY	1.7	16
ERK1/2	4ZZN	1.33	39
GRB2	3C7I	1.7	16
GSK3	1Q5K	1.94	29
HPH	4BQY	1.53	4
KEAP1	4IQK	1.97	3
MDM2	4OGN	1.377	11
MEK	3EQI	1.9	14
mTOR	3FAP	1.85	55
NAMPT	4O13	1.75	2
NQO2	1SG0	1.5	0
p53	2VUK	1.5	42
PARG	4B1H	2	1
PARP1	4ZZZ	1.9	8
PDE4B	4KP6	1.5	2
PDK1	5ACK	1.24	29
PGDH	2GDZ	1.65	0
PGES	4YL1	1.41	5
PI3K	4L23	2.501	41
PIN1	3I6C	1.3	7
PKC_alpha	4RA4	2.63	31
PLA2	3U8D	1.805	4
PP1A	3E7B	1.7	8
PP2A	2IE4	2.6	18
PPAR_gamma	3U9Q	1.522	19
PTEN	1D5R	2.1	32
Rac	1MH1	1.38	18
Raf	4XV9	2	13
Rap1a	4KVG	1.65	15
Ras	3K8Y	1.3	28
Rheb	3T5G	1.7	24
RhoA	1KMQ	1.55	19
S6K1	2Z7R	2	30
SGK1	3HDM	2.6	25
SIRT6	3ZG6	2.2	4
SOS	4NYJ	2.8522	18
TK receptor	4IBM	1.8	17
TNFR	1FT4	2.9	5
tyrRS	4Q93	2.1	1
ULK1	4WNO	1.56	10

#### Network-effect based compound clustering

To infer the network level effects of molecular docking on compound clustering, the generated docking scores were first rank normalised as explained in the methods section (Supplementary Table S4 sheet “Rank Normalisation”). Next, network topology parameter, node degree, calculated from PPI network (Table 1) was multiplied with the rank-normalised docking score (Box 1; Supplementary Table S4 sheet “Network-effect”). Eventually, compounds were clustered and visualised using dendrogram (Fig. 4c). Interestingly, with the addition of network dimension to the docking score-based compound clustering (Fig. 4c), a distinct difference to the structure-based and docking score-based clustering is observed (Figs 4a, b), further explained in next section.

### Emerging Insights on compounds based on the multi-dimensional deep screening pipeline

To highlight the impact using network-based compound clustering over similarity-based or docking-score based clustering, we present case studies for two pairs of compounds (1) rapamycin and vitamin C (ascorbic acid) and (2) retinol and retinoic acid.

#### Rapamycin and vitamin C

As shown in Fig. 2, both rapamycin and vitamin C have a different structure and size (Figs. 2 and 4a). Also, based on the docking scores they are clustered in different groups (Fig. 4b). However, after including the network parameter in the clustering algorithm, we could see that both rapamycin and vitamin C show similar network-level effects and are clustered together (Fig. 4c). It is worth noting that a distinct set of proteins associated with caloric restriction such as ADYCA, CAMKK2 and PDK1 were similarly affected by these two compounds. It is pertinent to note that the possibility of having a similar effect on a subset of cell-vitality-associated pathways was hypothesised based on the network-based clustering pipeline, although based on the docking score, these compounds can be inferred to have completely different structure and different MOA. The potential similarity between rapamycin and vitamin C at the network level provide testable hypotheses, which need to be investigated both at the mechanism level and in experimental assays. Although the biological interpretation of the results is beyond the scope of this study, rapamycin and vitamin C have been described as longevity drugs albeit different mechanisms seem to be involved.^52,78,79^

#### Retinoic acid and retinol

Retinoic acid and retinol are highly similar structurally (Figs 2 and 4a) and are grouped based on the docking scores (Fig. 4b). However, they were clustered in separate bins over network (Fig. 4c), suggesting a different mechanism of action for these two compounds. Interestingly, some studies have reported the differential effects of retinol and retinoic acid in human cells,^50,80–82^ thus further strengthening the impact of network-based compound screening pipeline.

The two examples analysed before show that we need to take into account all the information revealed by the different clustering methods. Thus, molecules with structural similarities could trigger different mechanism and this information is essential for the decision making process. In particular, it could allow identifying beneficial compound combinations that are not revealed by structural similarity or docking scores. For example, the combination of C-xyloside and vitamin C might have a broader effect on cell vitality and skin ageing parameters than the single compounds separately. Even though these two compounds are clustered together based on the docking scores, they cluster in separate bins at the network clustering level.

Box 1 Flow diagram to compute PPI network-effect in compound screening pipeline

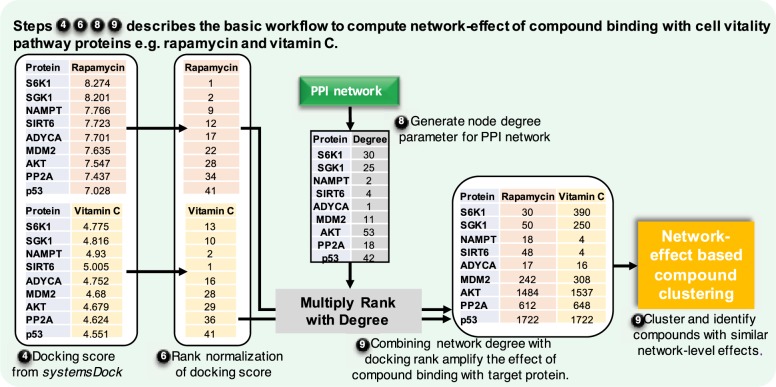

## Discussions

Here we introduce an original and innovative computational pipeline for compound assessment combining multiple dimensions of compound structure and docking profile to obtain deeper, emergent insights into the compound effect landscape. Further, to demonstrate the usability of the pipeline, we constructed a deep-curated, literature-driven molecular-level, mechanistic map for signalling and metabolic pathways associated with cell vitality, identifying and characterising the potential targets and proteins in the map. We further extended the pathway map to build a PPI network to capture the overall effects of the compounds. Using the protein targets identified by these specific pathways and networks, and applying the *systemsDock*^15,16^ framework to compute binding profiles for the compound list, the compounds were clustered on three dimensions—structure-similarity, binding profile and network effect. Comparative analysis of the clustering dimensions revealed the ability of our pipeline to identify new clusters of compounds that differ in structure or binding profile but may potentially have similar effect signature at the network level (rapamycin and vitamin C for example).

Structure-similarity is a well-known method to identify compounds with similar structures; however, the method is less robust in identifying biologically meaningful similarities as it lacks the information associated with compounds’ chemical properties or molecular interactions. By contrast, docking-score based clustering captures the molecular-level interactions between compounds and target proteins, but it does not capture the holistic effect of compound over a biological phenomenon as the technique is strictly limited by the availability of protein tertiary structure.

Network-effect based compound clustering overcomes these limitations of the former two approaches by (1) rank normalisation of docking score generating a comparable docking profile for each test compound and (2) combining PPI network topology with docking rank to indirectly incorporate the effects of proteins with no available tertiary structures. Both these features of network-guided clustering group compounds with similar holistic effect over a biological phenotype. At the same time, the differences in clustering between the docking-score and the PPI network for some compounds highlight the sensitivity of the clustering results to the network topology.

While the current pipeline elucidates the network-level effects of compound-protein interaction, the extent of phenotypical effects captured is dependent on the existing knowledge. The PPI network captures all possible interactions among proteins; however, it is possible that some of the interactions are not relevant for cell vitality thus changing the network effects on the compound clustering. Similarly, the coverage of the manually curated pathway map may miss specific interactions and thus bias the clustering results. Furthermore, these functional PPI networks provide only a qualitative measure of protein functionality within the network and do not infer on the abundance of each protein in the cell.

Therefore, the flexibility to modify, enhance and customise the pipeline is an important characteristic of building next-generation computational pipelines which can provide deep screening of compounds on multiple dimensions. For example in the future version of the pipeline, we plan to integrate publicly available protein tertiary structure prediction tools to predict the tertiary structure for proteins for which no PDB structures are available in the databases. Our current pipeline highlights the importance of multi-dimensional screen in capturing emergent properties of compounds which may not be apparent from a single-dimensional analysis using, for example, a high-throughput docking profile screen. At the same time, defining the boundaries of the dimensions in the pipeline, depending on data availability, focus areas of the compound effects and existing knowledge of the underlying biology, are some of the key issues in leveraging such pipelines. With the increasing availability of multi-omics data, the pipeline can be enhanced with powerful machine learning (including deep learning) techniques to identify unique features across the different datasets. This can further add value to the pipeline and its potential to enable deep screening of compound assessment across multiple domains.

## Methods

All methods associated with this study are part of the Results section.

### Reporting summary

Further information on experimental design is available in the Nature Research Reporting Summary linked to this article.

## Supplementary information

Supplementary Figures

Supplementary Table S1

Supplementary Table S2

Supplementary Table S3

Supplementary Table S4

Reporting Summary

## Data Availability

The authors declare that all data supporting the findings of this study are available within the paper [and its supplementary information files].
