# Supplementary material for: Multi-dimensional computational pipeline for large-scale deep screening of compound effect assessment: an in silico case study on ageing-related compounds
Source: NPJ Syst Biol Appl. 2019 Nov 26;5:42. doi: 10.1038/s41540-019-0119-y (PMC6879499; doi:10.1038/s41540-019-0119-y)
Supplement: Supplementary file 1 — Supplementary Figures [file 41540_2019_119_MOESM1_ESM.pdf]

Supplementary Figures

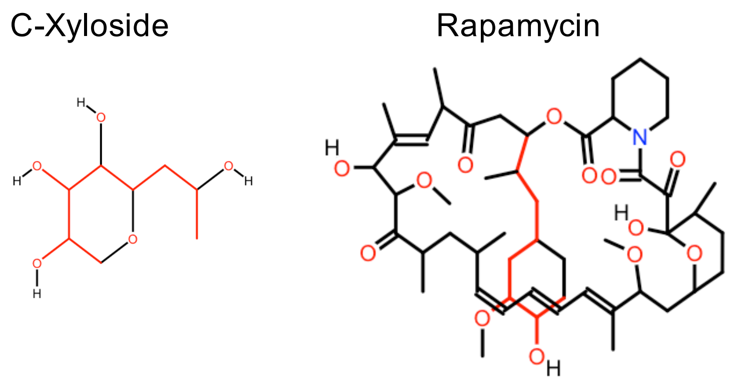

Supplementary Figure S1: MCS result between C-Xyloside and Rapamycin. Common substructure between the chemicals is highlighted in red.

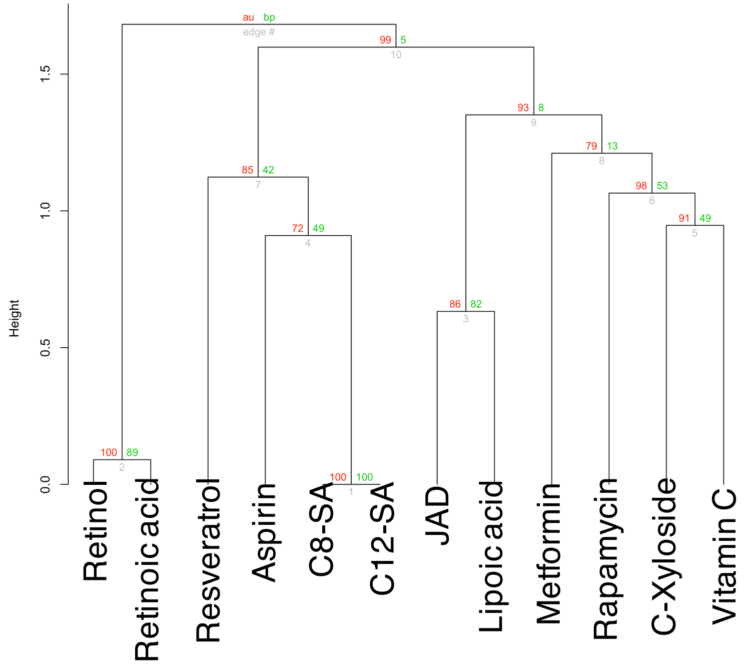

Supplementary Figure S2: Compound clustering dendrogram based on ECFPs.
